# Development of FRET‐based high‐throughput screening for viral RNase III inhibitors

**DOI:** 10.1111/mpp.12942

**Published:** 2020-05-21

**Authors:** Linping Wang, Jani Saarela, Sylvain Poque, Jari P.T. Valkonen

**Affiliations:** ^1^ Department of Agricultural Sciences University of Helsinki Helsinki Finland; ^2^ Institute for Molecular Medicine Finland University of Helsinki Helsinki Finland

**Keywords:** fluorescence resonance energy transfer, high‐throughput screening, RNA silencing suppressor, RNase III, small interfering RNA

## Abstract

The class 1 ribonuclease III (RNase III) encoded by *Sweet potato chlorotic stunt virus* (CSR3) suppresses RNA silencing in plant cells and thereby counters the host antiviral response by cleaving host small interfering RNAs, which are indispensable components of the plant RNA interference (RNAi) pathway. The synergy between sweet potato chlorotic stunt virus and sweet potato feathery mottle virus can reduce crop yields by 90%. Inhibitors of CSR3 might prove efficacious to counter this viral threat, yet no screen has been carried out to identify such inhibitors. Here, we report a novel high‐throughput screening (HTS) assay based on fluorescence resonance energy transfer (FRET) for identifying inhibitors of CSR3. For monitoring CSR3 activity via HTS, we used a small interfering RNA substrate that was labelled with a FRET‐compatible dye. The optimized HTS assay yielded 109 potential inhibitors of CSR3 out of 6,620 compounds tested from different small‐molecule libraries. The three best inhibitor candidates were validated with a dose–response assay. In addition, a parallel screen of the selected candidates was carried out for a similar class 1 RNase III enzyme from *Escherichia coli* (EcR3), and this screen yielded a different set of inhibitors. Thus, our results show that the CSR3 and EcR3 enzymes were inhibited by distinct types of molecules, indicating that this HTS assay could be widely applied in drug discovery of class 1 RNase III enzymes.

## Graphical abstract

A novel high‐throughput screening assay based on fluorescence resonance energy transfer was developed to identify inhibitors for RNase III.

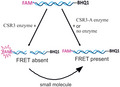

## 1 INTRODUCTION

RNA interference (RNAi) is an important defence‐response system of eukaryotic cells that results in the silencing of viral gene transcripts (Ratcliff *et al.*, 1997; Fire *et al.*, 1998). In the process, small interfering RNAs (siRNAs) play a crucial role in amplification and maintenance of the silencing response through loading onto Argonaute proteins, which uses siRNAs to target and cleave homologous viral RNAs (Chiu and Rana, 2003). As such, RNAi has become a powerful research tool for studies of gene function and siRNA‐based antiviral therapies (Tuschl and Borkhardt, 2002; Small, 2007; Badia *et al.*, 2017; Ghosh *et al.*, 2017; Qureshi *et al.*, 2018).

Viruses  are some of the most damaging pathogens in sweet potato, and more than 30 viruses in nine families have been reported to infect the crop. Among them, the crinivirus sweet potato chlorotic stunt virus (SPCSV) is one of the most harmful viruses because of its key role in synergistic infections (Clark *et al.*, 2012; Valkonen *et al.*, 2015). Studies have shown that SPCSV can build synergistic interactions with RNA viruses (potyviruses, ipomoviruses, carlaviruses, and cucumoviruses) as well as DNA viruses (cavemoviruses, begomoviruses, and solendoviruses), increasing plant susceptibility to those viruses and causing severe symptoms (Mukasa *et al.*, 2006; Untiveros *et al.*, 2007; Kreuze *et al.*, 2008; Cuellar *et al.*, 2011, 2015). Among them, the disease caused by the synergistic interaction of SPCSV and the potyvirus sweet potato feathery mottle virus (SPFMV) is the most common and devastating disease in sweet potato (Gutierrez *et al.*, 2003; Tairo *et al.*, 2005; Clark *et al.*, 2012). Furthermore, it has been proved that the synergistic interaction was mediated by a class 1 RNase III protein encoded by the RNA1 genome segment of SPCSV (abbreviated as CSR3 in this study), and its endonuclease function was necessary for its role as an RNA silencing suppressor in inducing synergistic disease (Kreuze *et al.*, 2005; Cuellar *et al.*, 2009; Weinheimer *et al.*, 2015). Therefore, considering the key role of CSR3, identification of CSR3 inhibitors could be an effective strategy to defend against synergistic virus disease in sweet potato.

Enzymes of the ribonuclease III (RNase III) family are expressed in a variety of organisms including bacteria and multicellular eukaryotes, with their enzyme size ranging from 140 to 1,900 amino acid residues (Court *et al.*, 2013). Among them, RNase III of SPCSV (CSR3) can cleave siRNAs, leading to the suppression of the RNA silencing pathway in sweet potato (Kreuze *et al.*, 2005). In addition, in viruses, another RNase III with similar function has only been found in the animal virus *Pike‐perch iridovirus* up to now (Weinheimer *et al.*, 2015). Studies have demonstrated that CSR3 sequences are highly conserved in SPCSV that infect wild species of sweet potato, indicating that CSR3‐mediated host RNAi suppression is a conserved function that is necessary for infectivity (Tugume *et al.*, 2013). Despite the important role of CSR3 in suppressing the host RNA silencing, no inhibitors for RNase III of SPCSV and *Pike‐perch iridovirus* have been reported, and no inhibitor screen has been applied for RNase III family enzymes according to the Binding Database (https://www.bindingdb.org/bind/index.jsp) or Web of Science (https://apps.webofknowledge.com). At present, several viral RNA silencing suppressors (RSSs) have been reported in plants, such as P19 of tombusviruses, HcPro of potyviruses, 2b of cucumoviruses, and P15 of pecluviruses, which interfere with different components of the RNA silencing pathway (Moissiard and Voinnet, 2004; Burgyan and Havelda, 2011). However, chemical inhibitor screening has been mainly carried out with proteins P19 and 2b, for example the screening of 5,000 chemicals for their ability to prevent siRNA binding to viral RSS of cucumber mosaic virus (CMV) (2b) and tomato bushy stunt virus (TBSV) (p19) led to the identification of strong inhibitors (Shimura *et al.*, 2008). Interestingly, this screening led to the identification of efficient antiviral agents against turnip mosaic virus (TuMV) later on (Fujiwara *et al.*, 2013). Inhibitor screening for the RSS was mainly carried out by methods such as surface plasmon resonance and electrophoretic mobility shift assay (Sagan *et al.*, 2007; Danielson *et al.*, 2015; Hu *et al.*, 2018).

Fluorescence resonance energy transfer (FRET), which is the nonradioactive transfer of energy between two molecules, has been used for studies of protein–protein interactions as well as high‐throughput screening (HTS) assays for drug discovery (Boute *et al.*, 2002; Benz *et al.*, 2011). FRET‐based assays are suitable for HTS because the method is sensitive and compatible with small volumes (Klostermeier *et al.*, 2004). FRET has also been used as a probe for tracking the stability, formation, delivery, and degradation of siRNAs in gene‐silencing studies by spectral imaging and a fluorescence detection system (Jarve *et al.*, 2007; Alabi *et al.*, 2012).

An effective HTS assay is necessary for screening libraries of compounds to identify candidate inhibitors of enzyme activities (Macarron *et al.*, 2011). Therefore, we developed a cost‐effective, FRET‐based HTS assay for identifying inhibitors of CSR3 using siRNA as the substrate. The coefficient *Z* prime (*Z*′), measuring statistical effect size as defined by Zhang *et al. *(1999), was employed to evaluate the suitability of the HTS assay by taking into account information from dynamic range and variation of signal measurements. Moreover, the decrease in reaction rate was defined in terms of the percentage of inhibition (PI) (Schultz, 2006). We used an siRNA tagged with a fluorescein reporter and a quencher as the substrate to measure the PI of compounds. Finally, our success with identifying CSR3 inhibitors with this HTS assay and its successful application on RNase III of *Escherichia coli* (EcR3) suggests that the HTS can be used for screening inhibitors of other CSR3‐like enzymes.

## 2 RESULTS

### 2.1 CSR3 characteristics

**FIGURE 1:**
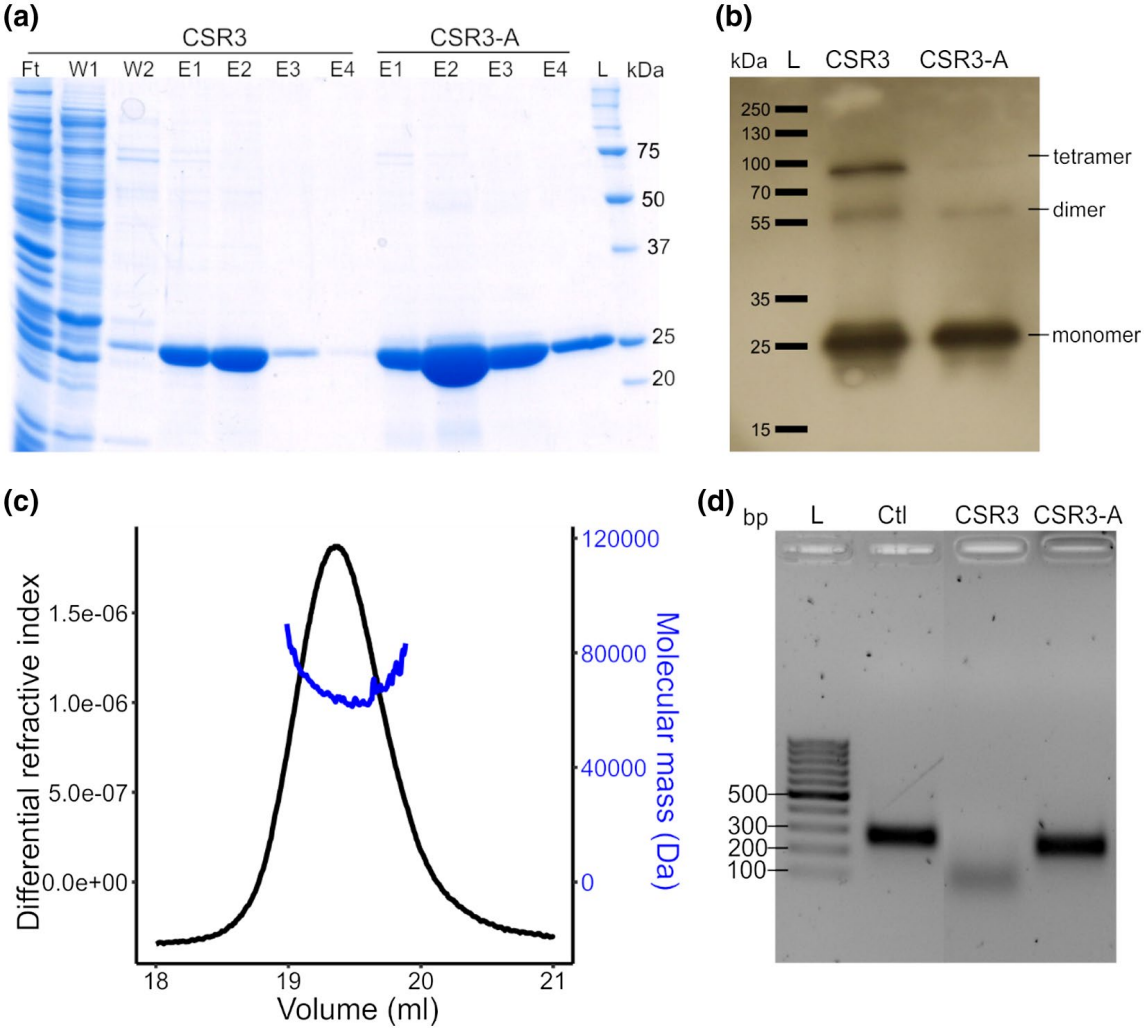
Preparation of CSR3 enzymes and characterization of catalytic activity and oligomerization. (a) SDS‐polyacrylamide gel electrophoresis of the purified CSR3 and CSR3‐A. The gel was stained with Coomassie Brilliant Blue; flow‐through (Ft), washing steps 1, 2 (W1, W2), elution 1–4 (E1–E4), protein ladder (L). (b) Western blotting for CSR3 and CSR3‐A using a rabbit polyclonal antiserum against CSR3. (c) Oligomerization of CSR3 was characterized by size‐exclusion chromatography coupled with multi‐angle light scattering. The calculated molecular mass was 68.93 kDa. (d) Agarose gel (2%) electrophoresis of the dsRNA substrate (200 bp) incubated at 37 °C for 45 min with CSR3, CSR3‐A, or no endoribonuclease (Ctl), DNA ladder (L)

Enzymes of high activity are necessary for the success of any HTS assay. For the development of our HTS assay, His‐tagged CSR3 and its double‐mutant CSR3‐A (D37A, D44A) were expressed in *E. coli* and purified with Ni‐NTA agarose. The recombinant CSR3 and its mutant were analysed with sodium dodecyl sulphate‐polyacrylamide gel electrophoresis (SDS‐PAGE), revealing a predominant band at c.26 kDa **(**Figure 1a). A second round of elution (Figure 1a) yielded the majority of CSR3, and aliquots of this fraction were made. In addition, western blotting showed that both proteins can exist as mixed monomer, dimer, and tetramer in storage buffer (Figure 1b). We also characterized the oligomerization of CSR3 by size‐exclusion chromatography with detection using multi‐angle light scattering. The only detectable protein peak at molecular mass 68.93 kDa was larger than that of the theoretical dimer of molecular mass 52 kDa, which could be explained either because most of our CSR3 preparation comprised a mixture of dimers and tetramers in phosphate‐buffered saline (PBS) running buffer, or by the nonspherical nature of the dimer, which could cause a disruption during size‐exclusion elution (Figure 1c). In general, the molecule state of CSR3 was consistent with previous characterization of CSR3 by native western blotting (Weinheimer *et al.*, 2014) and with functional and structural studies of another class 1 RNase III from *Aquifex aeolicus*, which demonstrated that the dimer is the catalytically active form of class 1 RNase III enzymes (Blaszczyk *et al.*, 2001). The CSR3 activity was evaluated using a 200‐bp double‐stranded RNA (dsRNA) substrate. This substrate was cleaved to smaller dsRNA fragments in the presence of CSR3 (Figure 1d) but remained intact in the presence of CSR3‐A, as was also the case for the control reaction lacking any endoribonuclease (Ctl). These results validated the activity of the purified CSR3 enzyme.

### 2.2 FRET assay setup

**FIGURE 2:**
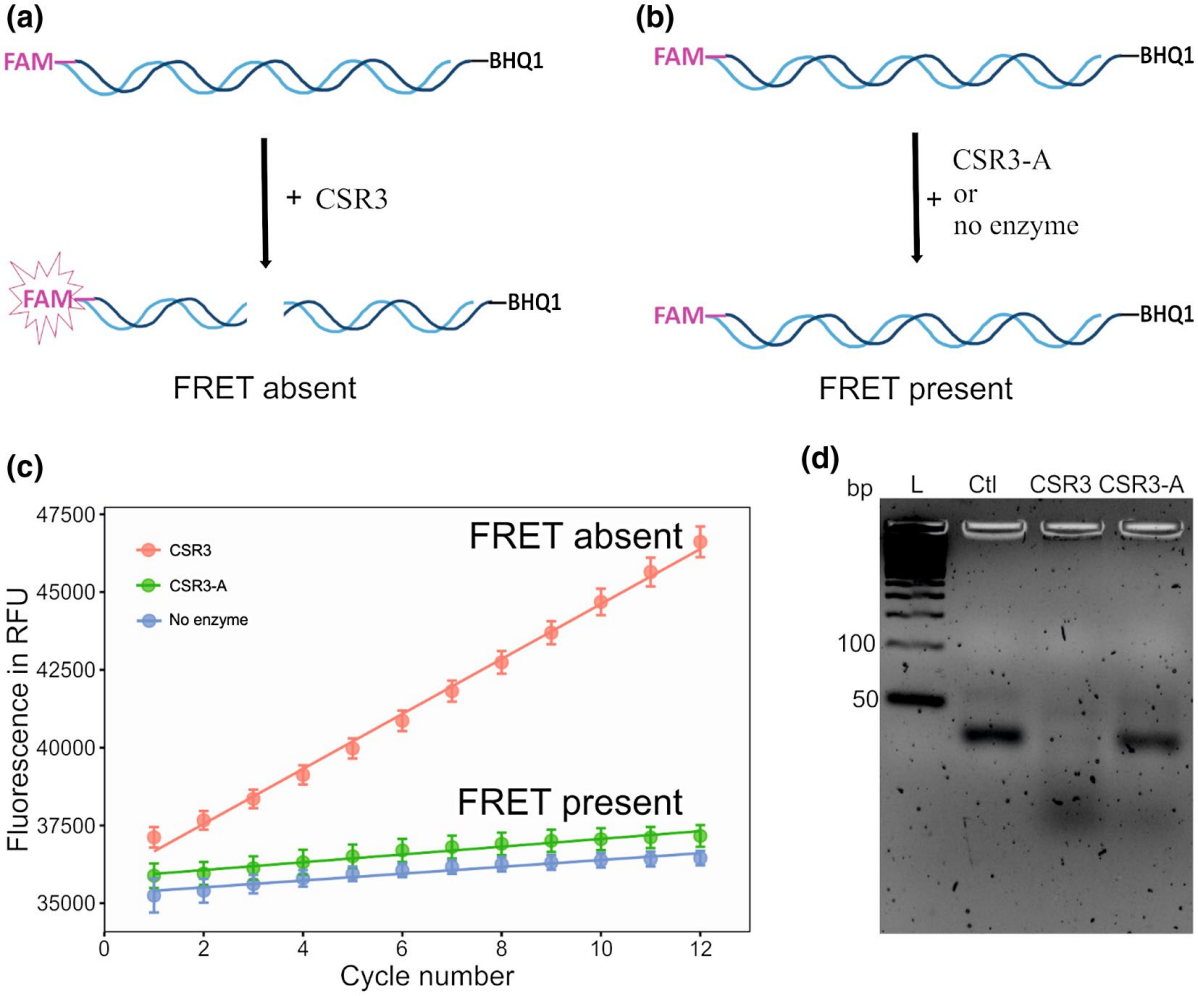
Overview of the fluorescence resonance energy transfer (FRET)‐based assay with CSR3 and CSR3‐A. Schematic representation of the FRET‐based assay. The labelled small interfering RNA (siRNA) was incubated with CSR3 (a FRET‐absent condition) or with either CSR3‐A or no enzyme (b FRET‐present condition). (c) A representation of fluorescence signal curve of the FRET‐absent and FRET‐present conditions, indicating by real‐time relative fluorescence units (RFU) in function of c.17 min total (12 cycles), measured at 37 °C by an optic module with excitation at 485 ± 6 nm and detection at 520 ± 5 nm. Data represent the mean ± *SD* (*n* = 24). (d) Agarose gel (2%) electrophoresis of labelled siRNA incubated for 30 min at 37 °C with CSR3, CSR3‐A, or without any enzyme (Ctl). All reactions contained 15 µl of 375 nM labelled siRNA. L, DNA ladder

Our HTS assay was based on FRET, in which CSR3 cleaves a labelled siRNA and generates a fluorescent signal **(**Figure 2a,b **)**. Specifically, a two‐nucleotide overhang of a 22‐bp siRNA was labelled with a fluorophore reporter (FAM) and a quencher (HBQ1) at the 5′ and 3′ ends, respectively, of the sense‐strand. Fluorescence was acquired with excitation/emission of 485/520 nm, reflecting the unquenched fluorescence intensity of the reporter. Two reaction conditions were used for the negative and positive controls to normalize CSR3‐inhibition data acquired for all compounds. For the negative control, cleavage of the labelled siRNA by CSR3 disrupts the energy transfer from the donor to the receptor and hence loss of FRET quenching, allowing us to detect the fluorescence emission of the reporter **(**Figure 2a). For the positive control (or blank control), the reaction was carried out either with the catalytically inactive CSR3‐A or in the absence of any added enzyme, so that labelled siRNA integrity was maintained and fluorescence emissions from the reporter remained quenched by HBQ1 (Figure 2b). There was a clear increase in fluorescence in the absence of FRET (i.e., negative controls), whereas fluorescence remained stable over time in the presence of FRET (i.e., positive controls) **(**Figure 2c). As both positive controls showed similar trends, in order to keep the HTS process simple no enzyme condition was applied as the positive control in the following optimization and screening assays. Moreover, these two control conditions were validated by analysis of the reaction products with 2% agarose gel electrophoresis **(**Figure 2d). These results indicate that the design of the assay is suitable for HTS.

### 2.3 CSR3 titration assay

**FIGURE 3:**
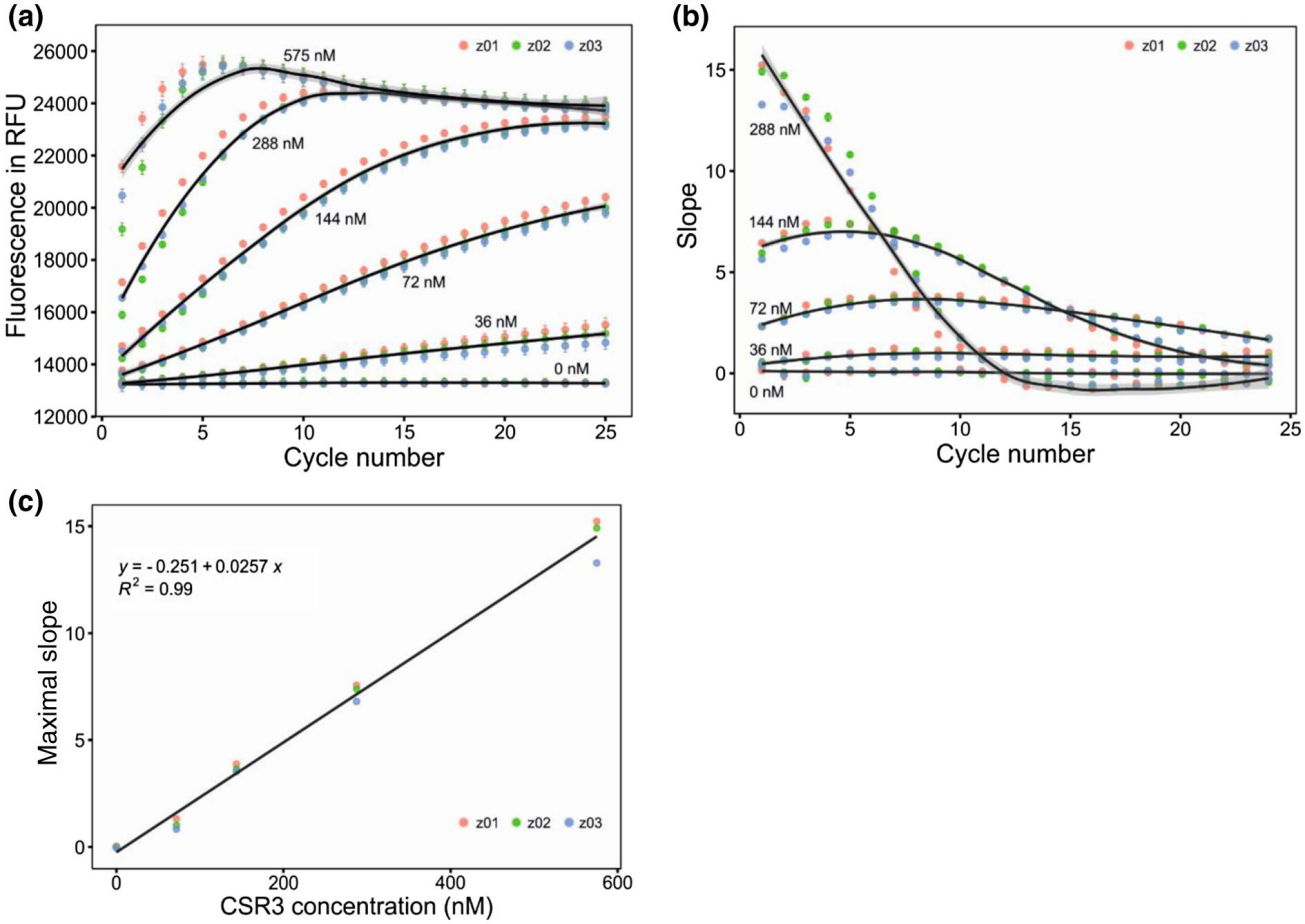
Titration assay with CSR3 and labelled small interfering RNA (siRNA). CSR3 (two‐fold dilution 575 to 36 nM, plus 0 nM control) and labelled siRNA (375 nM) were used to determine the optimal ratio of enzyme‐to‐substrate concentrations. Three replicate plates (z01, z02, z03) containing labelled siRNA were prepared with a dispenser, and enzyme was added to initiate the reaction. (a) Raw fluorescence expressed in RFU as a function of detection cycle. In total, 25 cycles were acquired (38 min total). Data represent the mean ± *SD* (*n* = 48). (b) Slope between neighbouring cycles obtained from raw fluorescence data (a). The highest CSR3 concentration (575 nM) was excluded. Data represent the mean ± *SD* (*n* = 48). (c) A linear correlation was found between maximal slopes obtained from neighbouring cycles (b) and CSR3 concentration

The rate of increase in fluorescence was dependent on the amount of enzyme and labelled siRNA in the reaction. Fluorescence measurements **(**Figure 3a) showed that, compared with a low concentration, a high concentration of CSR3 led to a rapid increase and higher fluorescence at the beginning of the kinetic measurement. To quantify the increase in fluorescence, we calculated the slopes (fluorescence changes over time in seconds depicting the reaction rate of CSR3) between all neighbouring detection cycles. The slopes were used to select the optimal detection time for each CSR3 concentration tested. The results suggested that the maximal initial slope for the concentrations 144, 72, and 36 nM of CSR3 occurred after 5, 10, and 14 reaction cycles, respectively (Figure 3b), indicating that a lower concentration of enzyme resulted in a longer period during which kinetics could be monitored. A very high kinetic rate was observed with 288 and 575 nM CSR3 concentrations during the first cycle (shown for 288 nM in Figure 3b), making it impossible to monitor the reaction at high enzyme concentrations, that is, the labelled siRNA was cleaved before fluorescence could be monitored. Furthermore, maximal slopes were linearly correlated with CSR3 concentration (Figure 3c), indicating that the initial velocity of the reaction correlated positively with CSR3 concentration with a fixed concentration of substrate. The *Z*′ values with five concentrations and three replicates (Table 1) suggested that an enzyme concentration ranging from 72 to 144 nM could be used because the resulting *Z*′ values were >0.9. Therefore, the final concentration of enzyme 100 nM CSR3 and substrate 375 nM siRNA were used in HTS assay. Moreover, these two control conditions indicated that both the labelled siRNA and enzyme were stable at room temperature (25 °C), an important aspect for HTS given that 20 assay plates were required for our screening and each of them has to be run with 12 detection cycles.

**TABLE 1:** Calculated Z′ values for the five different CSR3 concentrations used in the three replicates over 12 cycles

CSR3 (nM)	Z′		
	0 min	40 min	80 min
575	0.42	0.57	−1.76
288	0.89	0.75	0.88
144	**0.94**	**0.95**	**0.96**
72	**0.93**	**0.95**	**0.94**
36	0.64	0.77	0.63

### 2.4 Determination of the *K*_d_ value for CSR3

**FIGURE 4:**
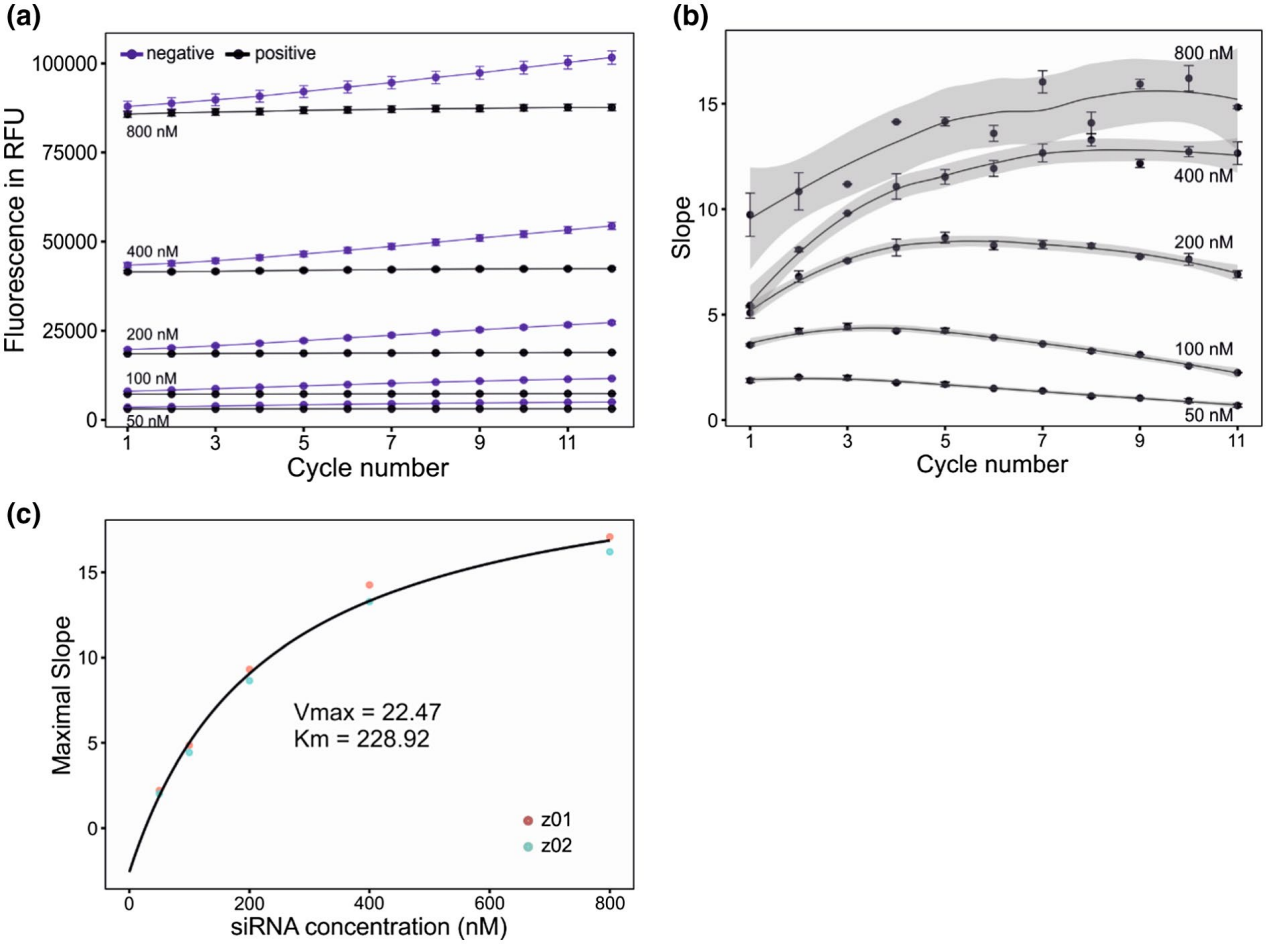
Determination of the *K*_d_ value for CSR3 and titration of labelled small interfering RNA (siRNA) substrates with 50 nM CSR3. (a) Raw fluorescence expressed in RFU as a function of detection cycles. In total, 12 cycles were acquired (c.17 min total). Data represent the mean ± *SD* (*n* = 24). Positive controls (lacking CSR3) was included for each substrate concentration. (b) Slope between neighbouring cycles obtained from raw fluorescence data (a). Data represent the mean ± *SD* (*n* = 24). (c) Maximal slope calculated from data obtained for five siRNA concentrations. The *K*_d_ value for CSR3 was calculated with the three‐parameter Michaelis–Menten model (MM.3) using the R package dcr, resulting in a *K*_m_ value of 228.92 nM

To monitor the kinetics of CSR3 endoribonuclease activity, two‐fold dilutions (yielding 50–800 nM) of the labelled siRNA substrate were tested with 50 nM CSR3. As expected, start points of raw fluorescence increased with the FRET‐siRNA concentration **(**Figure 4a). Moreover, the slope of the raw fluorescence between all neighbouring detection cycles was calculated for all cycle numbers. These data revealed that the maximal slope (2.2–17.1) increased with both FRET‐siRNA concentration (50–800 nM) and reaction cycles (2–10) (Figure 4b). Finally, the maximal slope for every labelled siRNA concentration was used to determine the catalytic rate of CSR3. Using the Michaelis–Menten model, the calculated *V*_max_ value was 22.47 and the *K*_m_ value was 228.92 nM **(**Figure 4c **)**. A similar kinetic study of an endonuclease activity demonstrated that FRET‐based methods were more sensitive and reproducible than gel electrophoresis‐based methods using radiolabelled substrates (Ghosh *et al.*, 1994).

### 2.5 Whole‐plate validation

**FIGURE 5:**
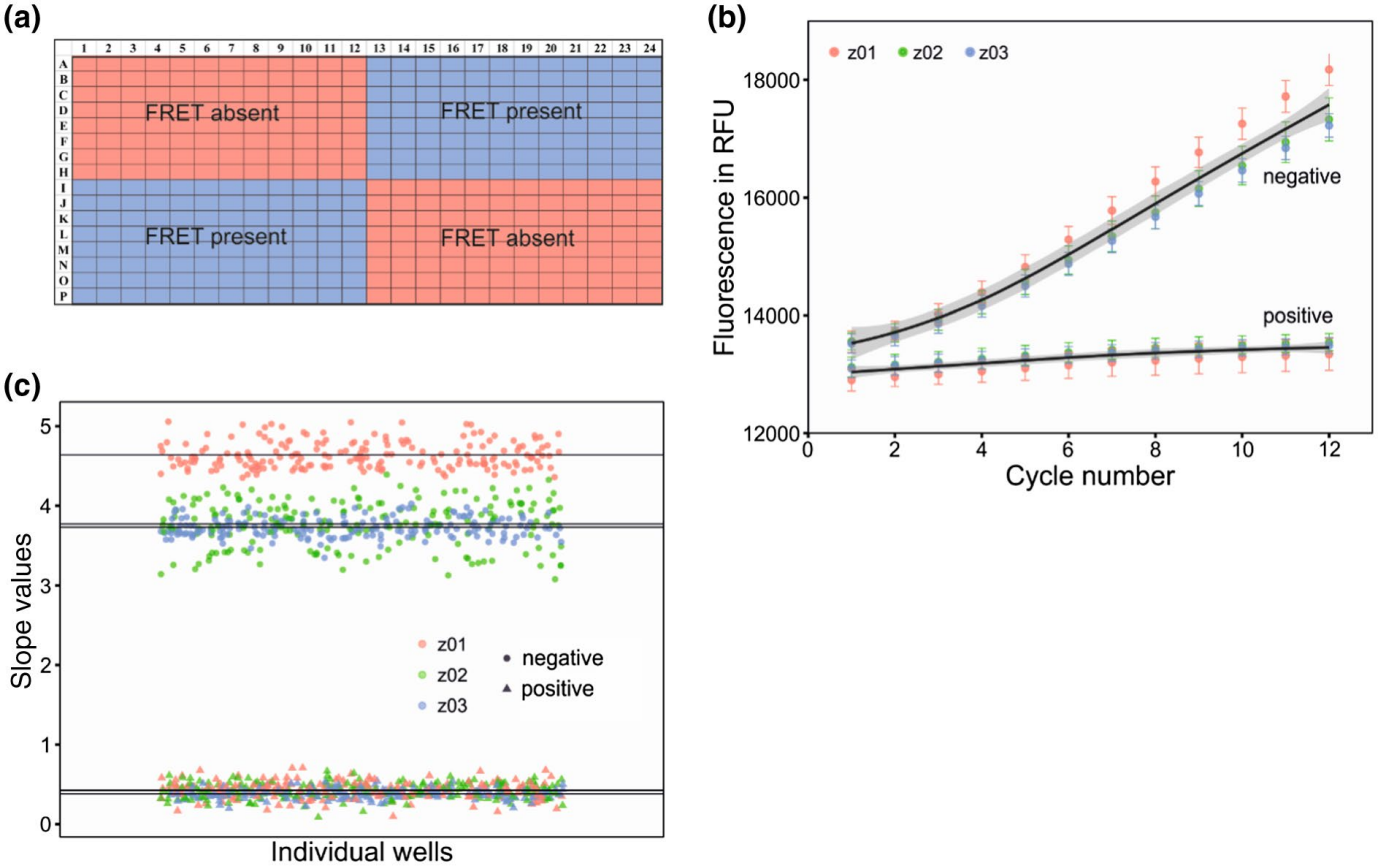
Whole‐plate assay to determine the homogeneity and consistency between plates for high‐throughput screening. (a) Whole‐plate layout, in which positive controls reflect the absence of fluorescence resonance energy transfer (FRET) (substrate cleaved) and negative controls reflect the presence of FRET (substrate not cleaved). (b) Raw fluorescence expressed in RFU as a function of detection cycle number reveals an obvious difference in fluorescence between the positive and negative controls. The test was carried out three times (z01, z02, and z03). Data represent the mean ± *SD* (*n* = 192). (c) Slope values obtained from raw fluorescence data for the three replicate plates (b) showing stable fluorescence in both controls. The mean slope values between negative and positive control are significantly different (analysis of variance, *p* < .001)

Homogeneity within and consistency between plates is crucial for HTS (Ahsen and Bomer, 2005). To assess these aspects of our assay, we used three replicate assay plates containing only positive and negative reactions **(**Figure 5a **)**. The raw fluorescence consistently increased over the 12 cycles for all three replicates, demonstrating the cross‐plate reproducibility of the assay **(**Figure 5b **)**. Fluorescence between the cleaved and uncleaved reactions could be easily distinguished. In the positive control (lacking CSR3), fluorescence remained essentially constant during the 12 cycles. In the negative control (containing CSR3), fluorescence increased linearly from cycles 4 to 12 and data obtained for this range of cycles were used to calculate the slope of the reaction. The slope values for the positive‐ and negative‐control reactions of the three replicates differed significantly (analysis of variance, ANOVA; *p* < .001; Figure 5c). By taking into account means of positive and negative slope values and their respective standard deviation, we estimated that *Z*′ values were >0.5 (0.77 ± 0.08) for all plates, whereas the signal‐to‐background ratio was 9.84 ± 0.75 and the signal‐to‐noise ratio was 43.17 ± 8.72 (see equation in section 4.8), indicating that our assay was suitable for HTS. To avoid any inaccuracy caused by variation between screenings, both negative and positive reactions were involved in each plate.

### 2.6 Primary HTS screen and a dose–response screen of CSR3

**FIGURE 6:**
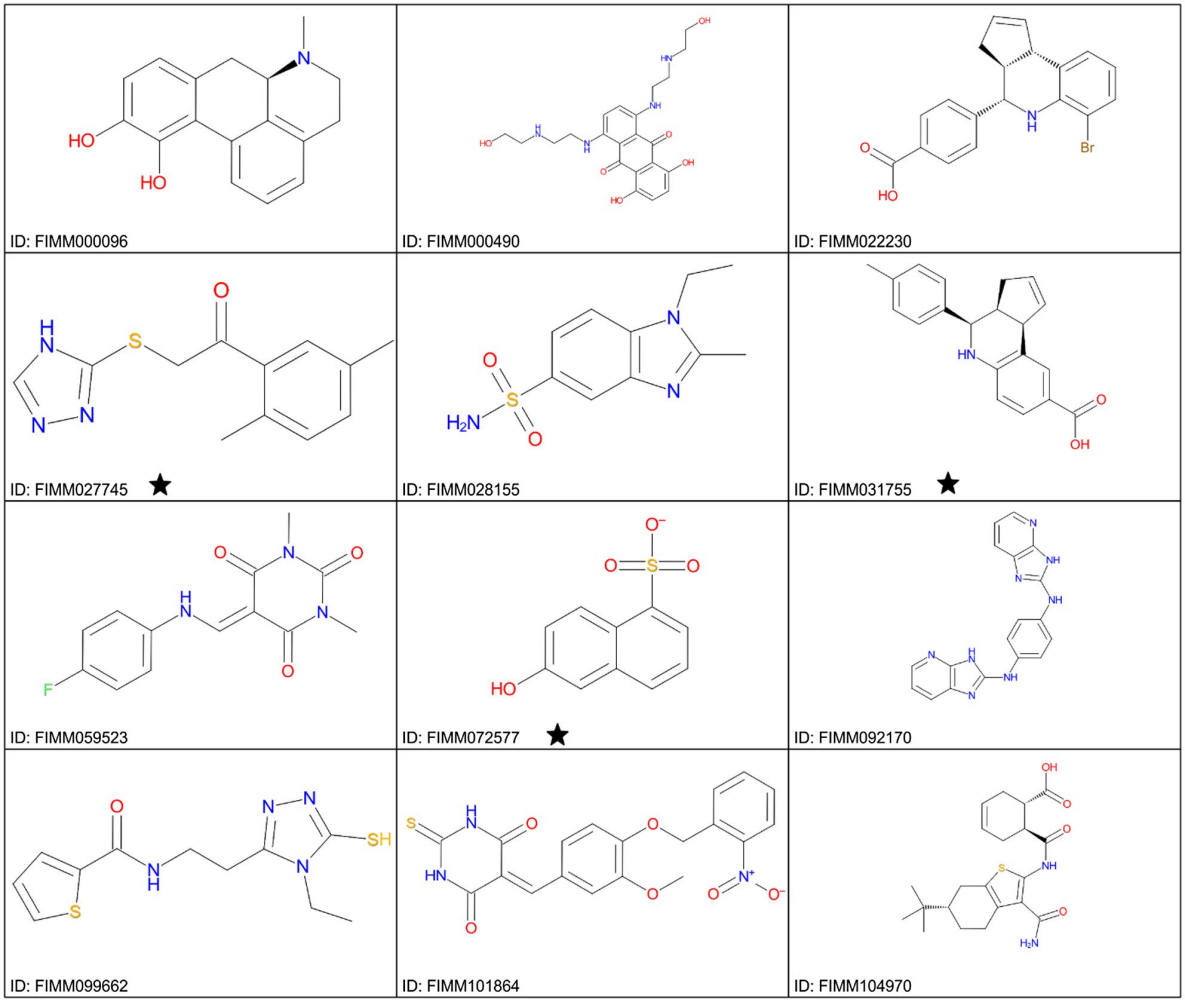
Structures of the compounds with percentage of inhibition (PI) > 90% in the primary screen. The black stars indicate the top three compounds identified by the dose–response screen

The FRET‐based assay was used in a primary screen of 6,620 small molecules of diverse structure. Of these 6,620 compounds, 109 (1.66%) had a PI > 30% (for PI distribution of those 109 compounds, see Table 2). The 12 compounds with a PI value >90% had diverse structures, and no common scaffold was readily apparent (Figure 6).

**TABLE 2:** Percentage of inhibition (PI) of assayed compounds

PI range (%)	≤30	31–40	41–50	51–60	61–70	71–80	81–90	91–100
No. of compounds	6,511	35	21	20	9	6	6	12

A dose–response assay was carried out with the top 109 compounds (concentration range 1.25 nM to 50 µM). The data were analysed with Breeze software, which generates dose–response curves and calculates the half‐maximal inhibitory concentration (IC_50_) and drug‐sensitivity score (DSS) for every compound. Considering that IC_50_ alone cannot comprehensively evaluate the drug sensitivity of the dose–response model in HTS assay, DSS was developed by Yadav *et al. *(2014) as a systematic algorithmic solution that integrated five factors: IC_50_, the slope at IC_50_, minimum activity level, and top and bottom asymptotes of dose–response model. The DSS values ranged from 0 to 19.2. Table 3 presents data for the top three compounds based on DSS values. The three structures differ (Figure 6, starred compounds). In addition, of the 12 compounds with PI > 90% in the primary screen, 10 were among the top 20 most potent compounds as determined in the dose–response screen, indicating that the assay was internally consistent even though a single CSR3 concentration was used. Table S1 lists the 109 compounds from the dose–response screens for CSR3, including information for compound identity, IC_50_, DSS, and dose–response curves.

**TABLE 3:** The top three most potent inhibitors of CSR3 as determined with the dose–response assay based on drug‐sensitivity score (DSS) values

Compound ID	FIMM027745	FIMM072577	FIMM031755
DSS	19.2	18.3	15.9
IC_50_	647.4 nM	699.3 nM	1,268.9 nM
Dose–response curves	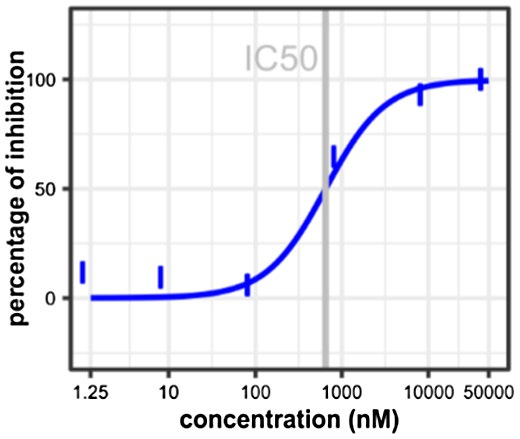	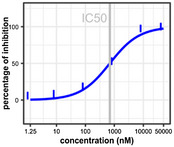	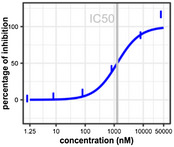

### 2.7 Screening of potential inhibitors for the EcR3

**FIGURE 7:**
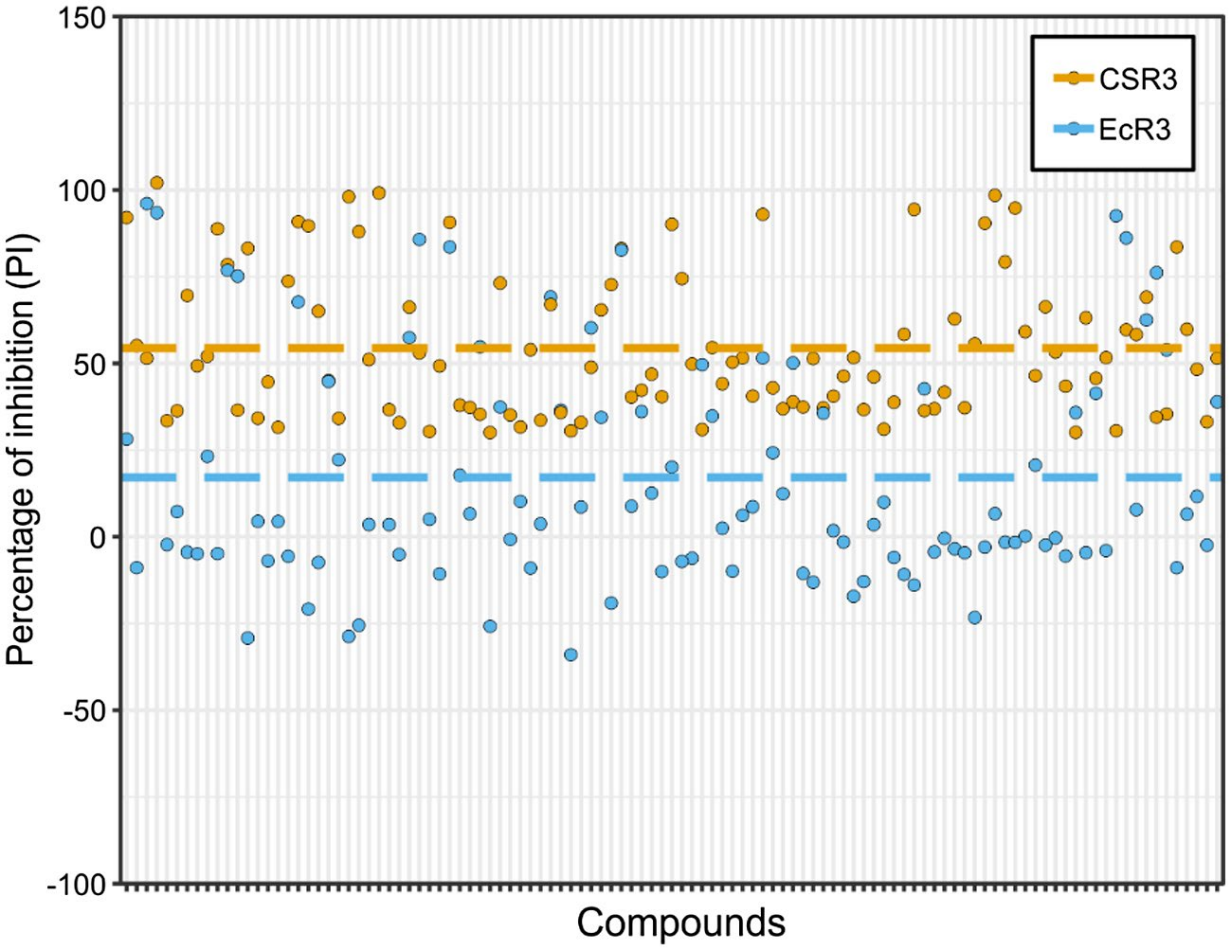
Comparison of percentage of inhibition (PI) values between CSR3 and EcR3. The PI values of 109 individual compounds for CSR3 of SPCSV (CSR3, light yellow dots) differed significantly (analysis of variance, *p* < .001) from those for RNase III of *Escherichia coli* (EcR3, light blue dots). The average PI value for CSR3 was 54.4% (light yellow dashed line), and that for EcR3 was 17.1% (light blue line)

The aforementioned 109 compounds were screened for their ability to inhibit the activity of the EcR3. The concentration of each compound was 10 µM (*Z*′ = 0.82)*.* To get some idea about how widely the HTS method can be used for other class 1 RNase III and the specificity of identified inhibitors, EcR3 was screened with the selected 109 compounds. Results showed that there was a significant difference in PI values between the CSR3 and EcR3 screens (ANOVA, *p* < .001), with mean PI values of 54.4% and 17.1%, respectively (Figure 7). For EcR3, 32 of the 109 compounds had PI ≥ 30%; this was expected because the 109 compounds were selected based on the CSR3 screen. The differences in PI values might be explained by differences in the active sites of the two enzymes, in that one amino acid is different between EcR3 and CSR3, which might be critical (amino acid sequence alignment and active site, Figure S1a,b, respectively). Specifically, the active site of CSR3 comprises four residues, namely E40, D44, N126, and E129 (black arrows, Figure S1a). The lone difference is D114 of EcR3, which corresponds to N126 of CSR3, and previous studies have shown that, at this position, aspartic acid (D) is prevalent in most RNase III family enzymes (Nicholson, 2014).

## 3 DISCUSSION

Several strategies could be applied in plant epidemiology to control plant viruses by taking into consideration virus–vector and virus–host interactions. To combat viral diseases such as sweet potato virus disease, the most common and effective strategy to date has been to select resistant sweet potato genotypes (Tairo *et al.*, 2005; Ngailo *et al.*, 2019). However, there are few sweet potato cultivars resistant to sweet potato virus disease in the field, especially in East Africa where there is a great demand for virus‐resistant sweet potatoes for subsistence farming. Additionally, resistance observed under experimental conditions may not be reproducible in the field (Okada *et al.*, 2017). It has been documented that CSR3 of SPCSV targets the host plant's RNAi system to cripple the antiviral defence system and increase sweet potato sensitivity to other RNA viruses. Because there is no effective means of counteracting sweet potato virus disease at present, we believe that the identification of an inhibitor of this harmful RNAi suppressor would open the way for new antiviral strategies for sweet potato. Hence, we carried out a small‐molecule screening project targeting CSR3.

Class 1 RNase III enzymes such as CSR3 only contain an RNA‐binding domain and a catalytic domain, whereas other classes of RNase III enzymes such as Drosha and Dicer have more complex structures, with several domains, allowing the processing of microRNAs of different lengths (Court *et al.*, 2013; Liang *et al.*, 2014; Nicholson, 2014). Thus, it was expected that the class 1 RNase III enzymes would not have any size selectivity in vitro and can process any size of dsRNA. In addition, because the amino acid identity between CSR3 and *Ipomoea* endogenous RNase III is very low (<5%), we believe there is a high probability of identifying CSR3‐specific inhibitors. We selected a 22 bp siRNA with a two‐nucleotide overhang along with a specific FRET reporter (FAM) and quencher (BHQ1) for four main reasons. First, studies have shown that small RNA duplexes are properly processed by CSR3 (Weinheimer *et al.*, 2014). Second, eukaryotic siRNAs have both a structure and size that are typical of viral siRNAs (21–24 bp with a two‐nucleotide overhang). Third, and most importantly, this size is within the range of the Förster interaction radius**,** allowing use in FRET‐based HTS. Finally, the fluorescence spectrum of 485/520 nm used in this assay is common in commercially available fluorometers. Moreover, in the HTS assay, to avoid false‐positive results caused by the degradation or disassembly of two strands of the siRNAs substrate, both reporter and quencher were fused on the sense siRNA strand. Thus, the FRET signal could only be induced when siRNA was cleaved by CSR3. Based on this, the identified potential inhibitors could bind within the active site of RNase III or bind to a specific location essential for siRNA loading into CSR3.

FRET is a distance‐sensitive method requiring a Förster interaction radius of less than 10 nm (Piston and Kremers, 2007). FRET has been commonly used with 20‐ to 25‐nucleotide‐long labelled oligonucleotide probes (Didenko, 2001; Ram *et al.*, 2008). Moreover, FRET‐based methods allow measurements of fluorescence changes in real time because the FRET signal decays exponentially in nanoseconds without excitation (Piston and Kremers, 2007). To use FRET in HTS, reproducibility is an important factor owing to the rate of FRET decay, which is highly sensitive to experimental factors such as pH, buffer reagents, and fluorescence quenchers (Piston and Kremers, 2007). To ensure the assay performed well and consistently within and between plates, we carried out a three‐plate test with only negative and positive controls. This test was necessary because small molecules are usually tested at a single concentration for primary‐inhibitor HTS (Ahsen and Bomer, 2005; Campagnola *et al.*, 2011; Ilouga and Hesterkamp, 2012). We report here an HTS assay of 6,620 compounds cherry‐picked from different compound libraries provided by the Institute for Molecular Medicine Finland (FIMM). The HTS screening was carried out for 7 hr using 384‐well plates and a 20 µl volume. Considering the sensitivity of the FRET‐based assay, it could be possible to use the 1,536‐well format and miniaturize the assay to 1–5 µl per well (Rodems *et al.*, 2002). Therefore, our method could be easily adapted to screen larger compound libraries to identify potential new drugs. On the contrary, detection time is influenced by many factors, for example the ratio of the concentration of the enzyme to substrate, reaction buffer, and reaction conditions. In our primary assays, measuring kinetics for eight cycles (11 min total) was sufficient for obtaining good results, yet, we monitored the reaction for 12 cycles (17 min total) to obtain more consistent results. It is noteworthy that the measurement time cannot be less than 4 min because of the high variability in reaction kinetics caused by heating the plate while acquiring results. We recommend monitoring the HTS assay for ≥10 min for the purpose of identifying RNase III inhibitors. Moreover, we tried to carry out HTS at c.25 °C, but the catalytic efficiency was rather low, and therefore HTS assay required more enzyme or time to achieve similar results than the recommended 37 °C condition.

We identified 109 CSR3 inhibitors in the assays with a PI value of 30% as a threshold. By raising the threshold, one is able to effectively reduce the number of potential false‐positives, and induce false‐negatives (Ilouga and Hesterkamp, 2012). The best three compounds (FIMM027745, FIMM072577, and FIMM031755), according to their DSS in the dose–response screening of CSR3, were all synthetic compounds. Moreover, all three compounds have not been reported as effective drugs according to Drugbank (https://www.drugbank.ca/). However, according to PubChem database, FIMM031755 (CID: 7,114,450) has been involved in an inhibitor screening assay for Cytokine/receptor Binary Complex of humans (4KC3_B) and was shown to be effective at micromolar concentrations. FIMM027745 (CID: 712,810) has been used in several inhibitor screens, for example HIV‐1 reverse transcriptase and human heat shock 70 kDa protein, but was inactive in all studies.

In the future, the promising candidates could be transferred to plants. Two compounds were tested at several concentrations (0.1 nM to 100 µM) on sweet potato plants coinfected with both SPCSV and SPFMV grown in medium. Preliminary results of the two compounds FIMM027749 and FIMM072436 with different PI values (88% and 41%, respectively) showed that FIMM027749 was able to down‐regulate virus accumulation of both SPCSV and SPFMV by two to three times, but without displaying a typical dose–response curve **(**Figure S2a,b**)**. Compound FIMM072436, on the other hand, had a mild impact on SPFMV but no impact on SPCSV **(**Figure S2a,b). Their effects on virus accumulation in plants were consistent with the PI values obtained by in vitro assay. At the same time, plant height over time was estimated and both compounds did not have any effect on plant growth (see plant picture in Figure S2c), suggesting that they did not interfere with plant development through nonspecific interaction with host factors including endogenous RNase III. Therefore, the chance of identifying inhibitors that could work in vivo should be high. Nonetheless, these data are still preliminary and more in vivo assays or biochemistry tests will be needed to develop the candidates as antiviral agents in the future.

The identification in a fish DNA virus, *Pike‐perch iridovirus*, of an RNase III protein with a similar RNAi suppression function (Weinheimer *et al.*, 2015) indicates that RNase III‐based viral suppression exists in both plant RNA virus (SPCSV) and animal DNA virus (*Pike‐perch iridovirus*), thus it is likely that more viral RNase III acting as RSSs are present in nature waiting for discovery. In addition, our HTS assay was also successfully applied to the screening of bacterial EcR3, validating that this assay could be adapted to other similar RNase IIIs. Moreover, the different screening results between CSR3 to EcR3 demonstrate not only the specificity of the screening method but also the possibility of identifying broad‐spectrum inhibitors for class 1 RNase III. Taken together, our findings provide possibilities for developing new antiviral strategies for sweet potato virus disease, and our HTS assay could be used to identify inhibitors of various class 1 RNase III enzymes.

## 4 EXPERIMENTAL PROCEDURES

### 4.1 Protein expression and purification

C‐terminal His‐tagged CSR3 (GenBank ADQ42569.1) and CSR3‐A (D37A, D44A) were expressed in *E. coli* from the plasmid pET11d (Kreuze *et al.*, 2005; Weinheimer *et al.*, 2015). Bacterial cultures were grown under selection with ampicillin (100 µg/ml) and chloramphenicol (25 µg/ml) at 37 °C for 2 hr. Recombinant proteins were induced by adding 0.1 mM (final concentration) of isopropyl β‐d‐1‐thiogalactopyranoside into the culture medium and growing for 4 hr. Bacterial cells were lysed in lysis buffer (10 mM imidazole) supplemented with a tablet of protease inhibitor cocktail (cOmplete ULTAR Tablets, mini, Roche) per 10 ml lysis buffer and 1 mg/ml lysozyme (Sigma‐Aldrich) while incubating for 2 hr on ice. Sonication (50% duty cycle, 4 × 15 s; Branson Sonifier [B15OR Cell Disruptor B15]) was used to additionally disrupt cells and degrade nucleic acids.

Ni‐NTA agarose gravity‐flow chromatography (Qiagen) was used to purify His‐tagged proteins. Bacterial extract was loaded onto polypropylene columns (Qiagen) and washed successively with wash buffer 1 (50 mM imidazole) and wash buffer 2 (70 mM imidazole). Bound proteins were eluted with elution buffer (500 mM imidazole). Lysis buffer, wash buffer, and elution buffer were made using the His Buffer kit from GE Healthcare. Fractions containing high concentrations of pure protein were collected, subjected to buffer exchange with a PD MidiTrap G‐25 (GE Healthcare), and finally diluted in storage buffer (20 mM MgCl_2_, 40 mM Tris‐HCl, 100 mM NaCl, pH 8.0, 5% glycerol). The Bradford colorimetric method (Protein Assay, Dye Reagent concentrate, Bio‐Rad) and a NanoDrop spectrophotometer (Thermo Fisher Scientific; 280 nm) were used for protein quantification. Coomassie Brilliant Blue reagent [10% (vol/vol) glacial acetic acid, 40% (vol/vol) methanol, 1% (wt/vol) Coomassie Brilliant Blue G] was used to visualize proteins in gels following SDS‐PAGE.

### 4.2 CSR3 activity assay as assessed with agarose gel electrophoresis

The dsRNA molecules (200 bp) were generated using the TranscriptAid T7 High‐Yield Transcription kit (Thermo Fisher Scientific). To precipitate dsRNA, samples were incubated with 2.5 M ammonium acetate on ice for 15 min and centrifuged at 10,000 × g for 15 min at 4 °C. After removing the supernatant, the pellet was washed twice with 70% ethanol, air‐dried at room temperature for 10 min, and resuspended in RNase‐free water (50 µl). To reanneal RNA as a double strand, samples were incubated at 95 °C for 10 min, 65 °C for 1 min, and room temperature before storage at −20 °C. RNase activity was tested in 20 µl reactions (20 mM Tris‐HCl, 50 mM NaCl, 10 mM MgCl_2,_ pH 8) containing 300 ng of 200‐bp dsRNA and 200 ng enzyme. Each reaction was incubated for 40 min at 37 °C before loading on a 1% agarose gel.

### 4.3 Western blotting

Proteins were resolved via Tris‐glycine SDS‐PAGE (12% polyacrylamide) and transferred to polyvinylidene difluoride membrane (GE Healthcare) by electroblotting. After SDS‐PAGE electroblotting, gels were stained with Coomassie Brilliant Blue to confirm that the majority of proteins were transferred. Each membrane was then incubated for 60 min in phosphate‐buffered saline (PBS; 137 mM NaCl, 2.7 mM KCl, 10 mM Na_2_HPO_4_, 2 mM KH_2_PO_4_, pH 8) containing 0.1% (wt/vol) Tween‐20 (PBS‐Tween) with 5% non‐fat dried milk and washed 3 × 10 min in TBS‐Tween at room temperature. Then, each membrane was incubated for 1 hr with a CSR3‐specific rabbit polyclonal antibody (Kreuze *et al.*, 2005) diluted 1:1,000 in PBS‐Tween containing 2.5% dried milk. After three washes with PBS‐Tween, each membrane was soaked for 1 hr with horseradish peroxidase‐conjugated anti‐rabbit IgG (Sigma‐Aldrich) diluted 1:5,000, and bands were visualized with the West Pico chemiluminescence development substrate (Thermo Fisher Scientific). Chemiluminescence was detected with X‐ray film (Roche) using an enhanced chemiluminescence kit from GE Healthcare.

### 4.4 Size‐exclusion chromatography coupled with multi‐angle light scattering

Size‐exclusion chromatography coupled with multi‐angle light scattering was used for characterizing oligomeric states of CSR3. Samples of purified recombinant CSR3 diluted in PBS were loaded onto a Superdex S‐200 10/300 GL (GE Healthcare) column at 0.5 ml/min with an HPLC system (Shimadzu) coupled with MiniDAWN TREOS multi‐angle light‐scattering detector, and Optilab rEX refractive index detector (Wyatt Technology). The injection volume was 100 µl per sample, and chromatography was carried out at 4 °C. The concentration of protein in the effluent was measured with a light‐scattering detector (493‐TS; RI instrument 686‐REX, 658 nm; UV instrument SPD‐M20A). Data were analysed with ASTRA 6 software (Wyatt Technology).

### 4.5 Labelled siRNA constructs

A two‐nucleotide, 3′‐overhang siRNA of 22 bp labelled with a FAM reporter and BHQ1 quencher (forward: 5′‐FAM‐CGUAGUGGAAGUGGGAGAGGTC‐BHQ1‐3′; reverse: 5′‐CCUCUCCCACUUCCACUACGTG‐3′) were synthesized by Metabion. Their identity and purity were verified by HPLC. The RNA oligonucleotides were dissolved in annealing buffer (6 mM PIPES pH 7.5, containing 60 mM KCl and 0.2 mM MgCl_2_) at a concentration of 100 µM, aliquoted in volumes ranging from 10 to 200 µl and stored at −20 °C. Before use, siRNA was diluted to 15 µM (200 ng/µl) with annealing buffer, incubated for 2 min at 93 °C, and cooled to room temperature (≥30 min).

### 4.6 FRET assay development

The HTS assay was designed to maximize siRNA cleavage by CSR3. We used a donor–quencher fluorogenic siRNA as the substrate for CSR3. To optimize the enzyme:substrate ratio, a CSR3 titration assay was carried out by mixing six different concentrations of CSR3 (0–1150 nM) with 375 nM labelled siRNA according to results obtained from a preliminary test. To assess CSR3 activity, five labelled siRNA concentrations (50–800 nM) were used with 50 nM CSR3. To test the reproducibility and precision of the assay, full plates containing only negative (50 nM CSR3) and positive reactions (lacking CSR3) were tested with 375 nM labelled siRNA. Enzyme and substrate were prepared separately at 2 × final concentration, and then 10 µl of each was dispensed into every well with an automated dispenser (MultiFlo FX with single‐channel RAD‐cassettes; BioTek). Plates were 384‐well black flat‐bottomed microplates (#3544, Corning).

### 4.7 HTS assay

For the HTS assay, plates (10 µM compound in each well) were prepared with an Echo liquid handler (Labcyte) at the FIMM (Institute for Molecular Medicine Finland) High‐Throughput Biomedicine unit. CSR3 and annealed siRNA were diluted to 200 and 750 nM, respectively, in reaction buffer (50 mM Tris‐HCl, 125 mM NaCl, 25 mM MgCl_2_, pH 8.0). CSR3 solutions were dispensed into the wells of plates (10 µl CSR3 per well). Plates were incubated at room temperature for 15 min with shaking (450 rpm). Subsequently, 10 µl substrate was dispensed per well. The final CSR3 and substrate concentrations were 100 and 375 nM, respectively. The dose–response assay with six compound concentrations (range, 1.25 nM to 50 µM) was carried out with the same conditions. Plates were sealed, centrifuged briefly, and then, immediately analysed with a PHERAstar FS (BMG Labtech) with a fluorescence‐intensity optic module (excitation at 485 ± 6 nm, detection at 520 ± 5 nm) for 12 cycles (c.17 min total) at 37 °C. All dispensing was done using the BioTek MultiFlo FX. All screening plates contained positive‐control wells (lacking CSR3) and negative‐control wells (25 nl dimethyl sulphoxide, vehicle), which were used as standards to calculate the PI of compounds.

### 4.8 Data analysis

The endoribonuclease activity of CSR3 was calculated with slope, representing fluorescence changes in function of assay time (s), using MARS Data Analysis software (BMG Labtech). In addition, the *Z*′ of the assay was calculated according to Equation 1 (Zhang *et al.*, 1999), which was used to evaluate the suitability of the method during assay development, optimization, and screening. Moreover, the signal‐to‐noise ratio and signal‐to‐background ratio were also used to evaluate the quality of each assay: signal‐to‐noise ratio $=\;\;\left(\mu_{c-}\;-\;\mu_{c+}\right)/\sigma_{c+}$ and the signal‐to‐background ratio $=\;\mu_{c-}/\mu_{c+}$.

(1) $$Z^{\prime }=1-\frac{\left(3\sigma_{c+}+3\sigma_{c-}\right)}{\left|\mu_{c+}+\mu_{c-}\right|}$$

where *μ_c_*_+_ is the mean of the positive control, *μ_c_*_−_ is the mean of the negative control, *σ_c_*_+_ is the standard deviation of positive control, and *σ_c_*_−_ is the standard deviation of the negative control.

The PI of each compound for CSR3 was calculated according to Equation 2. A PI threshold of 30% was used as the cut‐off value for one concentration of HTS. Furthermore, the dose–response curves of PI in function of compound concentration were evaluated with DSS according to Yadav *et al. *(2014).

(2) $$PI=100\left[1-\frac{\left(S_{s}-S_{c+}\right)}{\left(S_{C-}-S_{c+}\right)}\right]\%$$

where *S_c_*_+_ is the mean of the slope of the positive control, *S_c_*_−_ is the mean of the slope of the negative control, and *S_s_* is the mean of the slope of samples.

The kinetic constant (*K*_d_) for CSR3 was calculated using the three‐parameter Michaelis–Menten model (MM.3) included in the R package dcr (Ritz *et al.*, 2015). The statistical significance of differences between values was assessed with one‐way ANOVA using the aov function in the R package.

## 5 CONFLICT OF INTEREST

Patent application FI 20205392.

## AUTHOR CONTRIBUTIONS

L.W. contributed to planning the research, experiment performance, interpreting the data, and writing the draft. J.S. and S.P. were involved in experiment performance and review. J.V. was involved in reviewing and ensuring infrastructure. All authors read and approved the final manuscript.

## Supporting information


**FIGURE S1** Amino acid sequence alignment of CSR3 and EcR3 and the active‐site structure of CSR3. (a) The amino acid sequences of CSR3 and EcR3 were aligned using multiple alignment using fast Fourier transform (MAFFT). The black arrows indicate the four active‐site residues, namely E40, D44, N126, and E129. D114 of EcR3 corresponds to N126 of CSR3. Asterisks denote identical residues, single dots denote chemically similar residues, and double dots denote a single‐base change in the respective codon. (b) CSR3 organized in dimer contains two catalytic domains (cyan and yellow) and two substrate‐binding domains (green). The surface of the active site and binding‐site residues (represented by stick structures) are highlighted. The three‐dimensional structure was modelled using I‐TASSER (https://zhanglab.ccmb.med.umich.edu/I-TASSER/)

**FIGURE S2** Inhibitor validation assay in planta. Sweet potato coinfected with SPCSV and SPFMV were grown in a medium (Wang *et al*., 2019) supplemented with a serial concentration of each compound (0.1 nM to 100 µM) containing 0.1% of DMSO. In the control condition, coinfected plants were grown on a media supplemented with 0.1% of DMSO. After 28 days of growth, SPCSV and SPFMV viral accumulation was estimated by measuring the relative expression of coat protein of both viruses by quantitative reverse transcription PCR (RT‐qPCR). Methods of RNA isolation and RT‑qPCR are described in (Wang *et al*., 2019). Down‐regulation of SPFMV (a) and SPCSV (b) accumulation induced by each compound relative to control plants, which was represented by log_2_ fold change of their respective coat proteins expression. Values are mean ± *SE* (*n* = 2–3). (c) Plant images of coinfected sweet potatoes grown in medium supplemented with compounds or with 0.1% of DMSO (control), after 28 days

**TABLE S1** A list of 109 compounds tested on CSR3, with information of compound identity, IC_50_, DSS, and dose–response curves

## Data Availability

The data that support the findings of this study are available from the corresponding author upon reasonable request.
