# Supplementary material for: Development of FRET‐based high‐throughput screening for viral RNase III inhibitors
Source: Mol Plant Pathol. 2020 May 21;21(7):961–74. doi: 10.1111/mpp.12942 (PMC7280029; doi:10.1111/mpp.12942)
Supplement: Supplementary file 1 — FIGURE S1 Amino acid sequence alignment of CSR3 and EcR3 and the active‐site structure of CSR3. (a) The amino acid sequences of CSR3 and EcR3 were aligned using multiple alignment using fast Fourier transform (MAFFT). The black arrows indicate the four active‐site residues, namely E40, D44, N126, and E129. D114 of EcR3 corresponds to N126 of CSR3. Asterisks denote identical residues, single dots denote chemically similar residues, and double dots denote a single‐base change in the respective codon. (b) CSR3 organized in dimer contains two catalytic domains (cyan and yellow) and two substrate‐binding domains (green). The surface of the active site and binding‐site residues (represented by stick structures) are highlighted. The three‐dimensional structure was modelled using I‐TASSER (https://zhanglab.ccmb.med.umich.edu/I-TASSER/) [file MPP-21-961-s001.pdf]

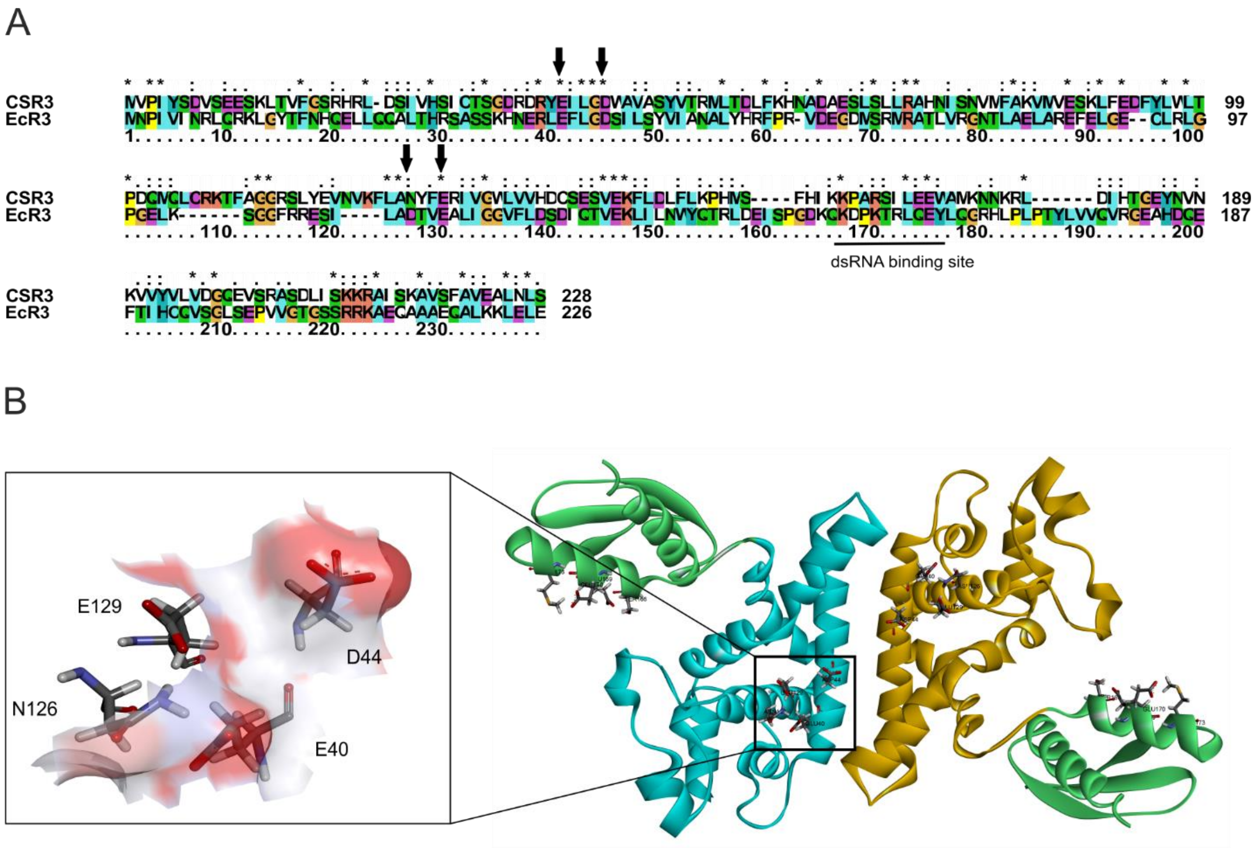

Fig. S1 Amino acid sequence alignment of CSR3 and EcR3 and the active-site structure of CSR3.

A. The amino acid sequences of CSR3 and EcR3 were aligned using MAFFT. The black arrows indicate the four active-site residues, namely E40, D44, N126, and E129. D114 of EcR3 corresponds to N126 of CSR3. Asterisks denote identical residues, single dots denote chemically similar residues, and double dots denote a single-base change in the respective codon. **B.** CSR3 organized in dimer contains two catalytic domains (cyan and yellow) and two substrate-binding domains (green). The surface of the active site and binding-site residues (represented by stick structures) are highlighted. The three-dimensional structure was modeled using I-TASSER (https://zhanglab.ccmb.med.umich.edu/I-TASSER/).
