# Supplementary material for: Development of FRET‐based high‐throughput screening for viral RNase III inhibitors
Source: Mol Plant Pathol. 2020 May 21;21(7):961–74. doi: 10.1111/mpp.12942 (PMC7280029; doi:10.1111/mpp.12942)
Supplement: Supplementary file 2 — FIGURE S2 Inhibitor validation assay in planta. Sweet potato coinfected with SPCSV and SPFMV were grown in a medium (Wang et al., 2019) supplemented with a serial concentration of each compound (0.1 nM to 100 µM) containing 0.1% of DMSO. In the control condition, coinfected plants were grown on a media supplemented with 0.1% of DMSO. After 28 days of growth, SPCSV and SPFMV viral accumulation was estimated by measuring the relative expression of coat protein of both viruses by quantitative reverse transcription PCR (RT‐qPCR). Methods of RNA isolation and RT‑qPCR are described in (Wang et al., 2019). Down‐regulation of SPFMV (a) and SPCSV (b) accumulation induced by each compound relative to control plants, which was represented by log2 fold change of their respective coat proteins expression. Values are mean ± SE (n = 2–3). (c) Plant images of coinfected sweet potatoes grown in medium supplemented with compounds or with 0.1% of DMSO (control), after 28 days [file MPP-21-961-s002.pdf]

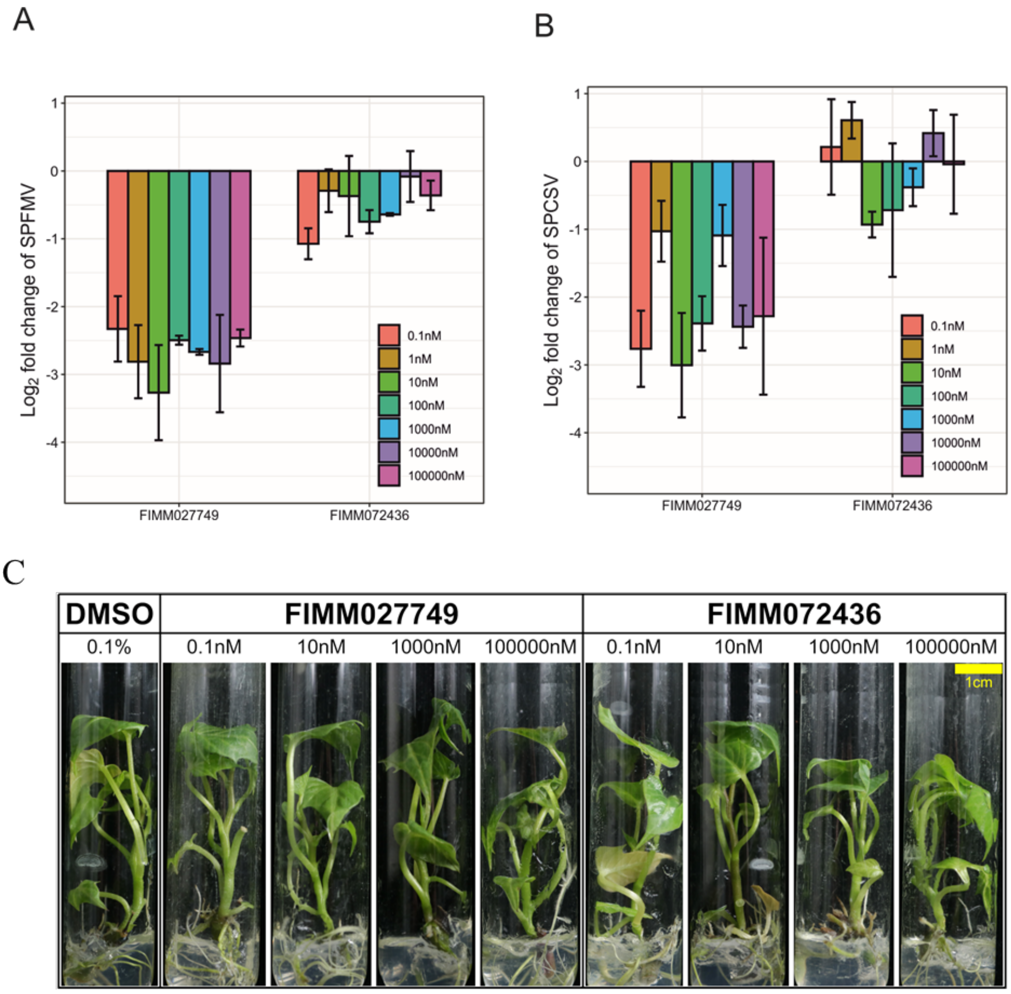

Fig. S2 Inhibitor validation assay *in planta*

Sweetpotato co-infected with SPCSV and SPFMV were grown in a medium (Wang *et al.*, 2019) supplemented with a serial concentration of each compound (0.1 nM to 100 µM) containing 0.1% of DMSO. In the control condition, co-infected plants were grown on a media supplemented with 0.1% of DMSO. After 28 days of growth, SPCSV and SPFMV viral accumulation was estimated by measuring the relative expression of coat protein of both viruses by RT-qPCR. Methods of RNA isolation and RT-qPCR are described in (Wang et al., 2019). Down-regulation of SPFMV (A) and SPCSV (B) accumulation induced by each compound relative to control plants, which was represented by log2 fold change of their respective coat proteins expression. Values are mean ± SE (n = 2-3). C Plant images of co-infected sweetpotatoes grown in medium supplemented with compounds or with 0.1% of DMSO (control), after 28 days.

Wang, L. P., Poque, S. and Valkonen, J. P. T. (2019) Phenotyping viral infection in sweetpotato using a high-throughput chlorophyll fluorescence and thermal imaging platform. *Plant Methods,* **15,** 116.
